# Mathematical Methods and Applications in Medical Imaging

**DOI:** 10.1155/2014/765163

**Published:** 2014-06-04

**Authors:** Liang Li, Tianye Niu, Seungryong Cho, Zhentian Wang

**Affiliations:** ^1^Department of Engineering Physics, Tsinghua University, Beijing 100084, China; ^2^Medical Physics Program, George W. Woodruff School, Georgia Institute of Technology, 770 State Street, Atlanta GA 30332-0745, USA; ^3^Department of Nuclear and Quantum Engineering, Korea Advanced Institute of Science & Technology, Yuseong-gu, Daejeon 305-701, Republic of Korea; ^4^Institute for Biomedical Engineering, University and ETH Zürich, 8092 Zürich, Switzerland

Medical imaging applies different techniques to acquire human images for clinical purposes, including diagnosis, monitoring, and treatment guidance. As a multidisciplinary field, medical imaging requires the improvements in both science and engineering to implement and maintain its noninvasive feature. Computational and mathematical methods are involved with imaging theories, models, reconstruction algorithms, image processing, quantitative imaging techniques, acceleration techniques, and multimodal imaging in medical imaging. The main purpose of this issue is bridging the gap between mathematical methods and their applications in medical imaging. This special issue covers most of the common medical imaging modalities, such as computed tomography (CT), magnetic resonance imaging (MRI), ultrasound, and various image processing methods, such as segmentation, enhancement, and registration.

This special issue has 23 papers which were reviewed by at least two reviewers. Six papers are involved with CT technique. One of the papers proves that the X-ray projection of the 3D mother wavelet is a 2D mother wavelet under certain conditions, which may be applied to 3D CT image processing. For accurate low-dose CT imaging, a practical method is presented by combining measurement-based scatter correction and compressed sensing-based iterative reconstruction in one paper. Another paper describes a CT imaging system with adjacent double X-ray sources. In one of the papers an imaging model is introduced to determine the best energy bin of the energy-discriminative photon counting detector for maximum material discrimination. Another paper presents an ASD-POCS-based optimization dual energy reconstruction algorithm with additional piecewise polynomial function constraint from the fully sampled low-energy data and undersampled high-energy data. In one paper, a so-called distributed alternating direction total variation minimization algorithm (Dis-ADTVM) is presented for few-view CT reconstruction. For multiecho T2-star weighted MRI image analysis, one paper presents an automatic mapping extraction algorithm to improve morphological evaluations in human brain. As a typical and important application, there are 13 papers involved with various medical image processing methods including segmentation, registration, enhancement, denoising, detection, and target location. In seven papers, 7 image segmentation methods are presented, respectively, by using different image features and mathematical methods, such as the symmetry technique, snake, multiscale technique, level-set, region growing, fuzzy C-means, graph cut, and random field. Two papers present two registration methods by using a Harris & SIFT features detection method and a coherent continuous formulation of multiple constraints, respectively. One paper introduces a mammographic image enhancement method by using multiscale transform and mathematical morphology. In another paper, a fast nonlocal means denoising method is proposed for 3D ultrasound speckle reduction based on the programmable graphic-processor-unit (GPU) hardware. One paper presents a nonnegative mixed-norm convex optimization method for mitotic cell detection in phase contrast microscopy. Another paper shows the evaluation results of the location of the mandibular canal and the thickness of the occlusal cortical bone at dental implant sites from the dental CBCT images. In addition there are 3 interesting papers on other mathematical applications in medical imaging. One of the papers shows the analysis and clinical evaluation of a single hemodialysis on phosphate removal of the internal fistula patients. Machine learning has been successfully applied in medical image processing even in image reconstruction. One paper provides an overview of recent support vector machine-based methods developed and applied in psychiatric neuroimaging. Another paper proposes a framework based on Quick Response (QR) coded watermark for patient authentication and controlled access to medical records in cloud-based teleradiology.

We hope that this special issue may represent the state of the art and would attract wide attention of the researchers in medical imaging field.

*Liang Li* *Liang Li* *Tianye Niu* *Tianye Niu* *Seungryong Cho* *Seungryong Cho* *Zhentian Wang* *Zhentian Wang*

